# Recommended care and care adherence following a diagnosis of Lynch syndrome: a mixed-methods study

**DOI:** 10.1186/s13053-019-0130-8

**Published:** 2019-12-16

**Authors:** Kathleen F. Mittendorf, Jessica Ezzell Hunter, Jennifer L. Schneider, Elizabeth Shuster, Alan F. Rope, Jamilyn Zepp, Marian J. Gilmore, Kristin R. Muessig, James V. Davis, Tia L. Kauffman, Kellene M. Bergen, Georgia L. Wiesner, Louise S. Acheson, Susan K. Peterson, Sapna Syngal, Jacob A. Reiss, Katrina A. B. Goddard

**Affiliations:** 1 Center for Health Research, Kaiser Permanente Northwest, 3800 N. Interstate Avenue, Portland, OR 97227 USA; 2 Northwest Permanente, Kaiser Permanente Northwest, Portland, OR USA; 3 Department of Medical Genetics, Kaiser Permanente Northwest, Portland, OR USA; 4 Vanderbilt Hereditary Cancer Program, Vanderbilt University Medical Center, Nashville, TN USA; 5 Case Western Reserve University, University Hospitals Cleveland Medical Center, Cleveland, OH USA; 6 The University of Texas MD Anderson Cancer Center, Houston, TX USA; 7 Dana-Farber Cancer Institute, Brigham and Women’s Hospital and Harvard Medical School, Boston, MA USA

**Keywords:** Lynch syndrome, Adherence, Hereditary Cancer, Risk reduction

## Abstract

**Background:**

Lynch syndrome (LS) is the most common hereditary colorectal cancer (CRC) syndrome. This study assesses trends in diagnosis of LS and adherence to recommended LS-related care in a large integrated healthcare organization (~ 575,000 members).

**Methods:**

Electronic medical record (EMR) data (1999–2015) were examined to identify patients with a diagnosis of LS. We examined their LS-associated care recommendations and adherence to these recommendations. Qualitative patient and provider interviews were conducted with the aim of identifying opportunities for improved care delivery.

**Results:**

We identified 74 patients with a diagnosis of LS; 64% were diagnosed with a LS-related malignancy prior to their diagnosis of LS. The time to LS diagnosis following development of a LS-related cancer decreased over time: before 2009 11% of individuals received a diagnosis of LS within 1 year of developing a LS-related cancer compared to 83% after 2009 (*p* < 0.0001). Colonoscopy recommendations were documented in the EMR for almost all patients with LS (96%). Documentation of other recommendations for cancer surveillance was less commonly found. Overall, patient adherence to colonoscopy was high (M = 81.5%; SD = 32.7%), and adherence to other recommendations varied. To improve care coordination, patients and providers suggested providing automated reminder prompts for LS-related surveillance, adding a LS-specific diagnosis code, and providing guidelines for LS-related surveillance in the EMR.

**Conclusions:**

We identified fewer than expected patients with LS in our large care system, indicating that there is still a diagnostic care gap. However, patients with LS were likely to receive and follow CRC surveillance recommendations. Recommendations for and adherence to extracolonic surveillance were variable. Improved care coordination and clearer documentation of the LS diagnosis is needed.

## Background

Lynch syndrome (LS) is the most common hereditary colorectal cancer (CRC) syndrome, accounting for about 3% of CRC cases [1]. Patients with LS are also at an increased risk for other malignancies such as endometrial, stomach, small bowel, and ovarian cancers [2]. LS is caused by pathogenic variants in DNA mismatch repair (MMR) genes [3–6], leading to accumulation of somatic alterations and tumor formation [7]. Research has shown that patients with LS have reduced life-expectancy [8], but CRC surveillance reduces risk of cancer and improves survival [9]. While guidelines for surveillance have evolved over time, current NCCN guidelines recommend annual or biannual colonoscopy, consideration for risk-reducing hysterectomy/bilateral salpingo-oophorectomy (BSO) or endometrial biopsy in women, and consideration for upper endoscopy, urinalysis, and other screenings in selected individuals [10]. While there have been efforts to improve LS diagnosis rates, less is known about the care patients receive after diagnosis.

Historically, family history criteria drove identification of patients with LS [3–6], but these criteria failed to detect all individuals with pathogenic genetic variants [11, 12]. Furthermore, diagnostic assessment for LS was not always performed even when individuals met criteria [13]. New universal tumor screening (UTS) programs assess all newly diagnosed CRC and endometrial cancers for MMR defects, improving detection of patients with LS [14, 15]. Outside of registries [16–18], few studies have followed patients for more than 1 year after diagnosis to assess electronic medical record (EMR)-documented patient adherence to colonoscopy [19, 20] These studies found that a personal history of CRC and receipt of genetic counseling were significant predictors of adherence [19, 20]. Further, few studies have explored adherence to LS-related surveillance and care beyond colonoscopy [21, 22].

A UTS program was implemented in our integrated healthcare organization in January 2016. However, in 2009 the Evaluation of Genomic Applications in Practice and Prevention (EGAPP) working group at the Centers for Disease Control and Prevention was the first to provide guidance on UTS programs, which may have increased awareness of LS and LS-related cancers. We evaluated whether this increased awareness would impact time-to-diagnosis of patients with LS within our integrated healthcare organization. We also evaluated what risk management guidance individuals diagnosed with LS received in our system and investigated their adherence to this guidance. Here, we utilized EMR data from an integrated healthcare organization to (1) examine the trends in time to diagnosis of LS from first cancer diagnosis, (2) determine the LS-related surveillance and care recommendations provided to patients and documented in the EMR, (3) quantify patient adherence to these EMR-documented risk management recommendations, and finally, we (4) used qualitative interviews with patients with LS from this population and their providers to identify strategies for improving adherence to LS-related care recommendations. This is the first study outside of registry populations to interrogate EMR-documented adherence to multiple types of LS surveillance. Combined with our qualitative data, these findings suggest promising interventions for closing existing clinical care gaps in patients with LS.

## Methods

### Study design

LS patients within our healthcare system were identified via diagnoses codes in the EMR, manual abstraction was used to determine all recommendations for LS-related care documented within the EMR, and patient adherence to these recommendations was examined using procedural code review. We conducted interviews with LS patients identified through this EMR interrogation process as well as with providers caring for this patient population.

### Setting

Kaiser Permanente Northwest (KPNW) is an integrated healthcare delivery system serving about 575,000 members in northwest Oregon and southwest Washington. KPNW membership is demographically representative of the region. This study was approved by the KPNW IRB, and a waiver of written informed consent was obtained to perform EMR review.

### Study population

All KPNW members with a high likelihood of having LS (*n* = 979) were identified by searching the EMR for potentially relevant diagnostic codes employed by the medical genetics department (Table 1). Under guidance of a genetics professional, we determined through this subset analysis that LS was unlikely in patients with codes of ICD-9 V83.89 or ICD-10 Z14.8 alone. Accordingly, we reviewed all charts with codes of ICD-9 V84.09 and/or ICD-10 Z15.09 (*n* = 117). Additional patients were identified by the medical genetics department (*n* = 3) or identified through a research study (*n* = 3; NCT01582841) [23]. Identified patients were included if EMR chart review confirmed LS diagnosis (*n* = 74). These patients were confirmed on the basis of specified genetic testing (*n* = 71), noted genetic diagnosis (*n* = 1), obligate carrier status (*n* = 1), or an early (1997) LS diagnosis and recommendations consistent with LS and a genetic diagnosis noted in a parent (*n* = 1); the earliest chart diagnosis date found was in 1997.

**Table 1:** Diagnosis codes used to identify patients with LS and their associated procedure codes

Diagnosis	ICD-9-CM	ICD-10-CM
Carrier of genetic disease	V83.89	Z14.8
Genetic susceptibility to or family history of cancer	V84.09	Z15.09
Procedure	ICD-9-CM^a^	CPT
Abdominal Ultrasound		76700, 76770
CA 125		86304
Colectomy	17.33–17.36, 45.73–45.76, 45.81, 45.82, 45.93	44140, 44141, 44152, 44204, 44310
Colonoscopy	45.21, 45.23, 45.24, 45.42, 48.23	G0105, G0121, 44388, 45300, 45305, 45331, 45378, 45380, 45381, 45385, 45386
EGD/Endoscopy	45.13, 45.16	43259, 43752, 43234
Endometrial Biopsy		57505, 58100
Pelvic Ultrasound		76856
Genetic Counseling		96040
Hysterectomy	68.3, 68.41, 68.49, 68.59	58150, 58180, 58260, 58571, 58573
Oophorectomy	65.61, 65.63, 65.64	58720, 58951, 58952
Transvaginal Ultrasound		76817, 76830, 76857
Urinalysis		81000–81003, 81015, 81099

^a^ICD-10-PCS codes were not yet instituted at our organization at the time of code extraction

### Manual chart abstraction

We developed a standardized data collection instrument to facilitate EMR chart review and curate data using Research Electronic Data Capture (REDCap) tools [24]. Medical genetics professionals conducted manual chart review and abstracted the following information, when available: LS diagnosis information (diagnosis date, tumor testing strategy and results, genetic testing results); personal cancer history (type of cancer, diagnosis date); family history; provider-documented recommended LS-related care and follow-up interval; history of total colectomy, hysterectomy, and oophorectomy.

### Electronic data collection

From the EMR, we identified procedures corresponding to LS-related surveillance. We first identified all procedure codes listed in the EMR for a subset of patients with LS diagnoses. The study team reviewed all codes to identify those matching documented care recommendations; 68 procedure codes, which mapped to 12 different procedure concepts (Table 1), were included in our final analysis. Surgical procedures were considered risk-reducing if they occurred after the diagnosis of LS *and* prior to the diagnosis of any cancer.

### Qualitative data collection

We recruited living patients currently receiving care within the KPNW health system from the patient cohort by mail and providers by email, with a follow-up telephone call or email for participation in qualitative interviews about LS; this same patient and provider cohort was used in Schneider et al., where additional methodological details are available regarding recruitment [25]. In brief, providers were recruited if they had at least one patient with a diagnosis of LS on their patient panel. We developed interview questions to assess LS care receipt, barriers to care, and advice for improvements. A trained qualitative interviewer (JS) conducted all interviews, which were audio-recorded and professionally transcribed. Patient interviews lasted 45–60 min; provider interviews lasted 20–30 min. Patients received a $20 gift card for participating; providers were offered no incentive, per KPNW policy. Qualitative interview themes related to patient recommendations for improving their ability to adhere to their LS-related recommendations are reported in this manuscript. Themes reported in Schneider et al. were compared to actual EMR-derived patient adherence data [25].

### Quantitative data and statistical analysis

For all patients with a LS-related cancer prior to their LS diagnosis, we compared the proportion of patients receiving their LS diagnosis within 1 year of first LS-related cancer diagnosis between: (1) patients first diagnosed with cancer between 1995 and 2008, and (2) patients first diagnosed with cancer in 2009 or later using the Mantel-Haenszel chi-square [26, 27]. These date ranges were selected to reflect the EGAPP 2009 recommendation for UTS, which we hypothesized would increase awareness of LS and LS-related cancers [28, 29].

To determine patient adherence to recommendations documented in the EMR, we defined recommendation intervals based on EMR notes (e.g., every year, every 2 years, etc.) for each individual and type of care. Intervals were designated on a per-calendar-year basis from the calendar year the recommendation was first documented through 2016. For each interval reviewed, we used EMR-documented procedures and events to classify each patient as adherent or not adherent to the recommendation. Intervals that were not observed to completion (e.g., the patient switched to a non-KPNW provider, the study period ended, an entire relevant organ was removed, or the patient died) were included in adherence analyses only if the recommendation was carried out by the patient during the observed portion of the interval. In the case of CA-125 and colonoscopy adherence, patients who were recommended to continue surveillance following BSO or colectomy, respectively, due to past cancer were retained in analyses. Descriptive statistics were computed for each patient’s adherence and for the cohort’s average adherence across all observed intervals.

To determine whether patients with a personal history of CRC had a different adherence rate to colonoscopy recommendations than patients with no history, a generalized estimating equation (GEE) approach was used to model history of CRC as a predictor of adherence (binary variable) across intervals while accounting for correlation of intervals observed per patient. Breaks in membership of any length and incomplete intervals (defined above) were removed from the analysis.

Quantitative analyses were performed using Microsoft Excel and Version 9.1 of the SAS System for Windows (SAS Institute Inc., Cary, NC, USA). A significance cutoff of α = 0.05 was set a priori. Adherence visualization as violin plots was performed using seaborn version 0.9.0 for python version 2.7.2.

### Qualitative Data Analysis

A team-member (JS) trained in open-coding techniques [30] coded the interviews using Atlas.ti Version 6.0 (Scientific Software Development GmbH, Berlin, Germany) and produced code reports for content analysis [31–33]. These reports were reviewed multiple times to determine initial themes, which the research team discussed and reviewed against the interview transcripts. Refined themes were reviewed in an iterative process until the research team reached consensus on interpretation. Here, we present the advice for improvements to care coordination; additional themes on barriers and facilitators to care identified from this analysis have been published elsewhere as aggregate data [25]. Additionally, we created a matrix to look at patient barriers, facilitators, and recommendations reported by these individuals on a per-patient basis alongside their specific adherence data.

## Results

Demographic data for the 74 KPNW patients identified with a chart-confirmed diagnosis of LS is presented in Table 2. Nearly two thirds (64%) of patients had a diagnosis of at least one LS-related cancer type prior to their diagnosis of LS. The remaining 36% of patients were diagnosed with LS because of family history of cancer or a known pathogenic variant in the family. Since KPNW did not institutionally implement UTS for all cases of CRC until January 2016, most of our cohort was identified following referral to the genetics department for evaluation. A clinical trial of UTS for patients receiving CRC surgery between January 2012 and December 2015 resulted in the initial identification and subsequent genetic diagnosis of 8 patients in our cohort.

**Table 2:** Characteristics of patients with LS at KPNW included in this study

Variable	N (%)^a^	
Sex		
Male	22 (30)	
Female	52 (70)	
Race/Ethnicity		
White/Non-Hispanic	44 (60)	
White/Hispanic	2 (3)	
White/Unknown	20 (27)	
Unknown/Hispanic	2 (3)	
Native American/Non-Hispanic	1 (1)	
Native American/Unknown	1 (1)	
Asian/Non-Hispanic	1 (1)	
Unknown/Unknown	3 (4)	
Genetic Alteration Status^a^		
*MLH1*	19 (23)	
*MSH2*	27 (37)	
*MSH6*	12 (16)	
*PMS2*	11 (15)	
*EPCAM*	5 (7)	
Genetic Alteration Type^b^		
Splicing Variant	11 (16)	
Missense	10 (14)	
Nonsense	15 (21)	
Frameshift	17 (24)	
Small deletion	1 (1)	
Promoter hypermethylatio	1 (1)	
Large deletion	16 (23)	
Number of Malignancies Preceding LS diagnosis		
One LS-Related Malignancy	37 (50)	
Two or more LS-Related Malignancies	10 (14)	
Type of LS-Related Malignancies Preceding LS diagnosis		
Colorectal Cancer	36 (49)	
Endometrial Cancer	10 (26)^c^	
Ovarian Cancer	5 (14)^d^	
Breast Cancer	5 (10)^e^	
Sebaceous Gland Skin Tumors	3 (4)	
Pancreatic Cancer	1 (1)	
Variable	Mean ± SD	Range
Age at LS Diagnosis	50.6 ± 13.9	19–79

^a^Percent is given out of number of patients with genetic status confirmed by chart-documented testing (*n* = 71). One patient was diagnosed on the basis of germline hypermethylation of the promoter of *MLH1* (included as *MLH1*). Two patients had alterations in more than one gene, with one being a deletion encompassing portions of both *EPCAM* and *MSH2*

^b^Percent is given out of number of patients with genetic alteration type accessible in the chart note (*n* = 70); one patient had two alterations with the alteration type documented (both missense); another patient had a single large deletion encompassing portions of two genes: *EPCAM* and *MSH2*. Large deletions were considered to be those greater than one exon that did not result in a frameshift

^c^Endometrial cancer rate defined as the number of women who had diagnosis of endometrial cancer prior to LS diagnosis by the number of women who had not had an unrelated hysterectomy prior to LS and endometrial cancer diagnoses (*n* = 38)

^d^Ovarian cancer rate defined as the number of women who had diagnosis of ovarian cancer prior to LS diagnosis by the number of women who had not had an unrelated BSO prior to LS and ovarian cancer diagnoses (*n* = 36)

^e^Breast cancer rate defined as the number of women who had breast cancer prior to LS diagnosis by the number of women (*n* = 52); no prior unrelated mastectomies were observed

Patients with a personal history of LS-related cancer were diagnosed with their first cancer between 1978 and 2015. For those patients, the time to the diagnosis of LS following their first cancer diagnosis appears to be decreasing. Only 2 of 18 (11%) patients diagnosed with their first cancer from 1995 to 2008 received a diagnosis of LS within 1 year (mean age at cancer diagnosis = 46 years, range 33–66 years), compared to 20 of 24 (83%) patients diagnosed with their first cancer after 2008 (mean age at cancer diagnosis = 55 years, range 34–76 years; *p* < 0.0001). Given that some patients had tumor screening through a clinical trial on UTS (*n* = 8, seven of whom who had their first LS cancer between 1995 and 2015), we repeated this comparison after removing these seven patients from the analysis. In this cohort, 2 of 17 (12%) patients diagnosed with their first cancer from 1995 to 2008 received a LS diagnosis within 1 year, while 16 of 18 (89%) patients diagnosed with their first cancer after 2008 received a LS diagnosis within 1 year (*p* < 0.0001).

Nearly all eligible patients with LS (96%) had documentation of a recommendation to receive colonoscopy surveillance at some point in their care. Of the three patients who did not have a recommendation for colonoscopy documented, one received genetic testing results after health plan membership ceased and one had obligate carrier status but refused genetic testing. Of the patients receiving an initial recommendation for colonoscopy, nearly all (93%) were advised to have colonoscopy at least as often as every 2 years.

The types and frequency of other surveillance recommendations documented for patients with LS were variable. Thirty-one to 69% of eligible patients ever received recommendations related to surveillance for gastric, urinary tract, endometrial, and ovarian cancer (Table 3). When recommended, the recommended interval length for endoscopic surveillance was notably variable among patients, including 1–2 years (27%), 2–3 years (35%), and 3–5 years (31%), and other intervals (6%).

**Table 3:** Patient adherence data for recommendations related to LS in 74 LS patients

Recommended Screening Procedure	Patients Eligible for Screening^a^ N	Patients Ever Receiving Recommendation N (% of total)^b^	Patients Observed^c^ N (% of recommended)	Patient Adherence N (% of observed)				Average Observed Intervals (per patient) M ± SD	Average Adherence (patient) % ± SD	Total Intervals N	Intervals Met N (%)
				0% adherence	> 0- < 50% adherence	50- < 100% adherence	100% adherence				
Colonoscopy (total cohort)	74	71 (97)	65 (92)	6 (9)	2 (3)	12 (18)	45 (69)	2.5 ± 2.0	81.5 ± 32.7	159	127 (80)
*Colon* *Cancer*	*37*	*36 (97)*	*31 (86)*	*3 (10)*	*0 (0)*	*6 (19)*	*22 (71)*	*2.9 ± 2.5*	*83.2 ± 31.9*	*91*	*78 (86)*
* No Colon* * Cancer*	*37*	*35 (95)*	*34 (97)*	*3 (9)*	*2 (6)*	*6 (18)*	*23 (68)*	*2.0 ± 1.4*	*80.0 ± 33.8*	*68*	*49 (72)*
Genetic Counseling	74	54 (73)	46 (85)	1 (2)	10 (22)	10 (22)	25 (54)	3.2 ± 3.0	73.5 ± 32.5	146	71 (49)
Endoscopy	74	51 (69)	33 (65)	6 (18)	1 (3)	7 (21)	19 (58)	1.9 ± 1.3	70.3 ± 39.5	63	41 (65)
Urinalysis	74	48 (65)	48 (100)	8 (17)	8 (17)	17 (35)	15 (31)	3.6 ± 2.0	57.7 ± 36.3	173	90 (52)
Abdominal Ultrasound	74	9 (12)	9 (100)	2 (22)	1 (11)	4 (44)	2 (22)	6.2 ± 3.8	49.4 ± 38.3	56	25 (45)
TVUS	31	12 (39)	11 (92)	6 (55)	3 (27)	1 (9)	1 (9)	4.0 ± 3.0	19.9 ± 31.3	48	10 (21)
CA-125	31	12 (39)	11 (92)	1 (9)	0 (0)	6 (55)	4 (36)	3.5 ± 2.7	72.6 ± 31.4	42	31 (74)
Endometrial Biopsy	29	9 (31)	9 (100)	1 (11)	5 (56)	3 (33)	0 (0)	6.6 ± 3.4	36.8 ± 29.8	59	21 (36)
Pelvic Ultrasound^d^	29	NA (NA)	8 (NA)	0 (0)	5 (63)	2 (25)	1 (13)	7 ± 3.3	41.2 ± 34.5	56	20 (36)

Abbreviations: TVUS, transvaginal ultrasound

^a^Discrete recommendations are annually, every 1–2 years, every 2–3 years, or every 3–5 years. Recommendations abstracted as “other options” were not included in adherence analysis

^b^Total patients with the relevant organ, except for colonoscopy, which was compared to all patients regardless of colectomy status

^c^Patients counted as observed if at least one interval was met, or at least one complete interval was observed to completion but not met. Patients with other interval options were also not included, due to the necessity to compare patients to a discrete interval

^d^Patients who received pelvic ultrasound were the same patients recommended to receive biopsy, but recommendations regarding pelvic ultrasound were not recorded. These patients were compared to their endometrial biopsy recommendation

**Fig. 1:**
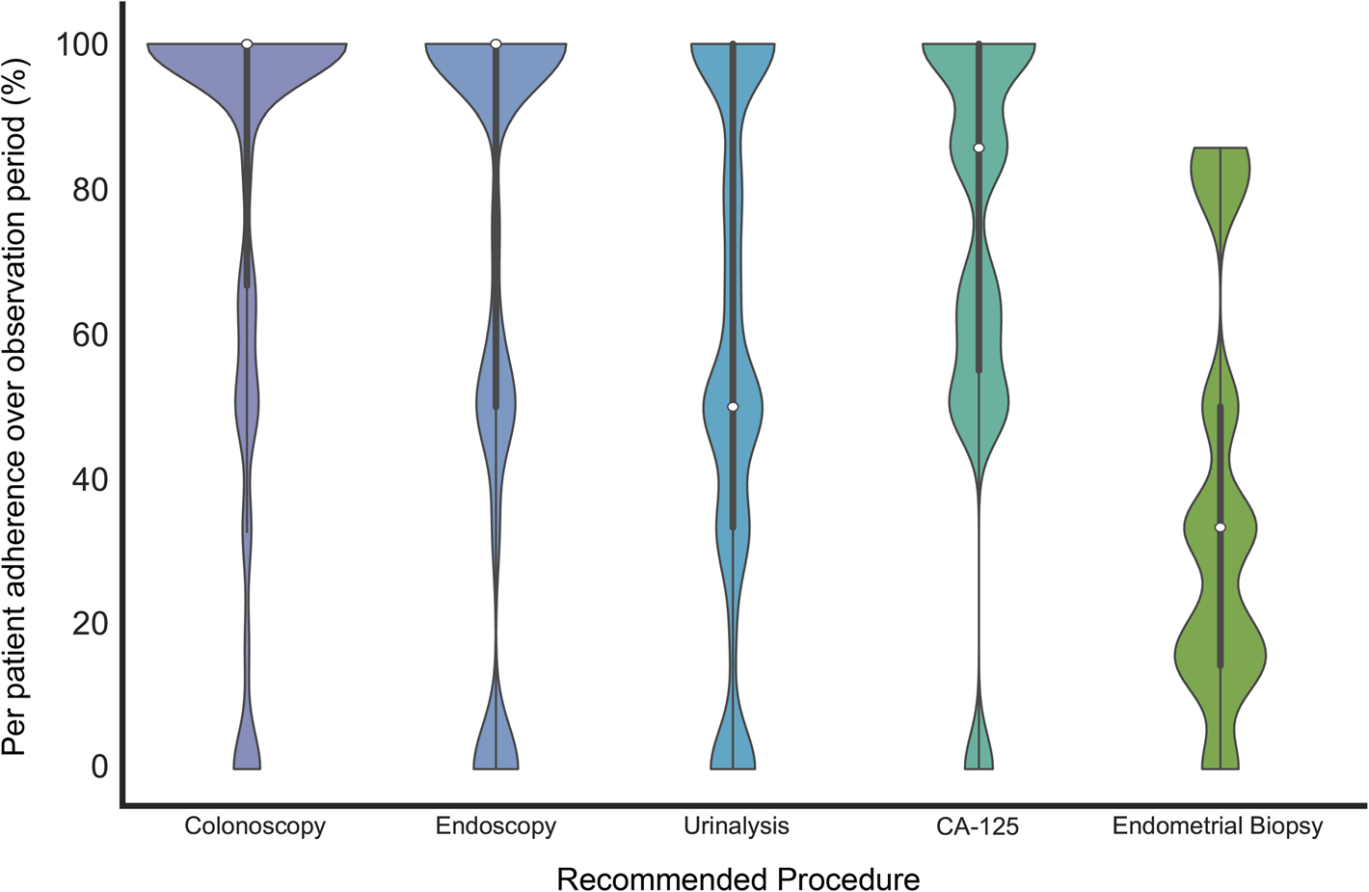
Patient adherence rates to LS-related care recommendations vary by recommendation type. Adherence rates for selected recommendations from Table 1 are depicted as violin plots. Thick grey bars indicate interquartile range (IQR), white dots represent the median, and the thin grey line represents the data distribution with the exception of points deemed outliers (points that are 1.5 x IQR above or below the upper and lower quartiles, respectively). On each side of the grey line is a kernel density estimation indicating the distribution shape of the data; distributions were truncated at the minimum and maximum value observations including outliers. Wider sections represent a higher density of observations near that value. Due to the large and consistent standard deviations for each recommendation type, a kernel scale factor of 0.15 was used for kernel density smoothing of each violin plot to enhance resolution; kernel size was determined programmatically by multiplying the scale factor by the standard deviation of the data within each bin. The number of observations and the standard deviations used in each plot are provided in Table 1

Adherence to recommended care by patients who received surveillance recommendations ranged from 19.9% (SD = 31.3%, transvaginal ultrasound; TVUS) to 81.5% (SD = 32.7%; colonoscopy). Perfect (100%) adherence to recommendations was also variable; no patients were 100% adherent to endometrial biopsy recommendations while 70% of patients were 100% adherent to colonoscopy recommendations (Table 3; Fig. 1). Adherence to colonoscopy on a per-interval basis was not significantly different between those with a history of CRC (86%) and those with no prior CRC diagnosis (73%, *p* = 0.1571).

In addition to surveillance recommendations, 73% of patients received a recommendation to contact medical genetics for any changes to their LS-related surveillance and care recommendations, as guidelines can change over time. Beyond their initial recommendations, only nine (12%) of the 74 patients had updates to their LS-related care and surveillance recommendations documented in their chart by any provider at some point following diagnosis of LS.

Consistent with KPNW policy, 100% of patients who were diagnosed with LS as a KPNW member visited medical genetics at least once. Seventeen patients were diagnosed with LS outside of KPNW; of those 17 patients, six (38%) sought counseling through KPNW medical genetics at some point after becoming a KPNW member. Of patients recommended to return to genetics with sufficient follow-up time (two or more intervals observed; *n* = 25), only six followed up with medical genetics only during the first interval and one never visited medical genetics; the remainder (*n* = 18; 72%) returned to genetics during at least one other interval.

Of the 52 women in the sample, many had BSO (40%) and/or hysterectomy (44%) prior to or concomitant with their diagnosis of LS, sometimes concurrent with a cancer diagnosis. Of those with an oophorectomy before diagnosis of LS (*n* = 21), five (24%) had had the surgery to treat or confirm/identify ovarian cancer. Of those who had a hysterectomy before diagnosis of LS (*n* = 23), nine (39%) had had the surgery to treat or confirm/identify endometrial cancer. A subset of women who had not had a prior BSO (*n* = 31 with at least one ovary) or hysterectomy (*n* = 29 with a uterus) elected to have risk-reducing surgery (BSO, 39%; hysterectomy, 41%) at some point after diagnosis with LS. None of these women were diagnosed with ovarian or endometrial cancer upon surgery. Two women received a diagnosis of endometrial cancer following their LS diagnosis, and had subsequent hysterectomy. Among the women who had not yet had both BSO and hysterectomy (*n* = 19), 47% were under 45 years of age at time of chart abstraction.

We completed 12 interviews with patients and 10 with providers. Demographic and clinical details about this sample have been published elsewhere [25]. Both patients and providers offered actionable suggestions that could address the observed variability in recommendations and the lack of care updates (Table 4). The most common recommendation from both patients and providers was automated care reminders for patients and providers to ensure screenings are scheduled at recommended intervals. Providers also recommended changes to the EMR to improve documentation of LS diagnosis and accessibility of surveillance recommendations. Additionally, patients recommended regular outreach from medical genetics, improvements in provider knowledge about LS and related surveillance recommendations, and more transparent care coordination practices when transitioning from their acute LS-cancer related treatment back to primary care.

**Table 4:** Provider and patient recommendations derived from qualitative interviews (*N* = 22; 10 Providers, 12 Patients)

Provider Recommendation	*N* = 10 (%)	Illustrative Quotes
Automated prompting of patient and PCP regarding recommended LS surveillance, including return to medical genetics	10 (100)	*“Make it similar to BRCA1… a patient of mine was recommended to get an MRI, alternating by six months. There was a helpful message that said, ‘hey it is time to order this patient’s MRI’. So that is great, thank you for providing me that support. So, it feels to me for Lynch Syndrome, there has to be that sort of mechanism.”* *“It would be great if patients would get a postcard every couple of years saying, ‘hey, you have Lynch Syndrome and you haven’t checked in with the genetics department for a couple of years - maybe you should come by.”*
Clear, consistent documentation of LS diagnosis available in EMR	10 (100)	*“So, something like that if it’s in the problem list, it says, ‘she has Lynch Syndrome, and she has had a history of this, and this is the recommendation for follow-up screening’. And that would really help, so every time I pull up the patient - anybody who pulls up the patient - it will be on their problem list and it will be clear.”*
Development of LS registry or improved coordination with other cancer/genetic registries	6 (60)	*“I think there needs to be some registry for who has these patients so that they can be followed regularly and it’s not falling on the PCP to do a panel search to try and figure out which one of their patients have Lynch Syndrome and did they have their colonoscopy recently.”*
Development of clinical resource practice guidelines for LS embedded within the EMR that highlight current surveillance recommendations and provide “steps” for providers making LS-related referrals	5 (50)	*“I think for the newer clinicians, and for those of us that have been here longer, there should be a practice guideline, clinical resource practice guideline for Lynch Syndrome that could have brief [information] on what we do and what the current recommendations are - a place that you could essentially update when there are new recommendations on how these patients are managed…I’d want it to be on my referral to genetics.”*
Offering refresher education course on LS and surveillance needs to all pertinent providers	5 (50)	*“I would love that [refresher class]. I mean, Lynch Syndrome is such a rare thing, you don’t come across it that often…I think it’s a field that can change a lot. I think it totally makes sense because even if you have only one patient, it’s good to know more about it.”*
Patient Recommendation	*N* = 12 (%)	Illustrative Quotes
Consistent, automated reminder prompts	9 (75)	*“I think it might be worthwhile for patients to be prompted on an annual basis for the appointment with either their primary care provider or their genetic counselor to go over all the screenings that they’re due for and to help them get those scheduled. I just think if you really want people to do it, it might be worth an extra follow-up to make sure people do it.”*
Regular outreach from genetics	5 (42)	*“I go on the Lynch Syndrome International [website] every once in a while, but you really have to search around these sites to find latest articles and stuff. So if genetics really wanted to be proactive, they could have somebody who is kind of finding that information and sending it out to us, rather than kind of having us research it… I’d just like a little bit of assistance.”*
Improve provider knowledge about LS and reasons for different surveillance recommendations	5 (42)	*“I think it would be good to make sure that providers, because they have varying degrees of knowledge, establish a base level of knowledge about Lynch that everybody has or figuring out what primary care doctors are treating patients who have Lynch and give them more information.”*
Proactively offering genetic screening for all cancers	4 (33)	*“The only thing I can say is it would have been nice, when you go over a patient’s family history and there is some history of colon cancer like in my case, it would be nice to find out earlier whether one has a gene mutation rather than waiting until a cancer developed.”*
Ensuring clarity regarding who is the primary care coordinator for LS-related care	2 (17)	*“Sometimes it’s a question about who’s in charge… it be helpful to have a better understanding of when the transition is being made, let’s say back from a specialist to your primary care person, who is the main person to be working with? Because sometimes, I wasn’t sure who was in charge.”*

Our matrix of patient reported recommendations and surveillance barriers identified from interviews alongside actual EMR-derived adherence for these patients (Table 5) provides further insight. Several patients [8–10, 12] were less than 100% adherent to their recommended colonoscopy interval. Two of these [9, 12] reported challenges to colonoscopy completion due to outcomes from their colon cancer (e.g., colostomy bag or removal of colon). One patient [10] reported their PCP encouraged discontinuing frequent colonoscopies given the patient’s older age, even though the patient expressed opposition to this suggestion. Another (patient 8) did not have a colonoscopy during their surveillance interval at KPNW despite reporting colonoscopy completion in qualitative interviews. However, that patient was diagnosed outside of KPNW, 5 years prior to receiving recommendations in our care system, and had another health condition that may have taken priority to colonoscopy while observed in our system. Some of the same patients were also less adherent to their recommended endoscopy interval, with two patients [8, 10] reporting lack of clarity on whether the endoscopy was needed or not, and if so, how to best coordinate it with their colonoscopy interval. Two other patients [1, 12] reported verbal communication from a provider indicating the endoscopy either was not needed any longer [1] or could be pushed out to a 5-year interval [12]. Several of these patients that demonstrated less than 100% adherence offered similar recommendations for improvement, including increasing provider knowledge about LS and related surveillance activities and receiving some form of pro-active support from their health care system, whether that be in the form of automated reminders or regular communication from the genetics department.

**Table 5:** Comparison of qualitative interview data to colonoscopy, endoscopy, and genetics visit adherence

	Patient 1	Patient 2	Patient 3	Patient 4	Patient 5	Patient 6	Patient 7	Patient 8	Patient 9	Patient 10	Patient 11	Patient 12
Patient Recommendations / Advice for Health System												
Automated reminders	Yes	Yes		Yes	Yes	Yes	Yes	Yes		Yes	Yes	
Desire regular outreach from genetics dept.		Yes		Yes		Yes		Yes			Yes	
Improve provider (PCP) knowledge of LS				Yes		Yes		Yes	Yes	Yes		
Ensure clarity regarding who is primary care coordinator	Yes	Yes										
Surveillance Tracking / Monitoring												
Patient takes sole responsibility		Yes				Yes				Yes	Yes	
Patient and providers jointly track/monitor	Yes		Yes	Yes	Yes		Yes	Yes	Yes			Yes
Patient Identified Barriers												
Patient as expert for colonoscopy frequency					Yes	Yes		Yes				
Colonoscopy frequency and prep burdensome	Yes				Yes				Yes			
Finding knowledgeable providers (PCP) or seeing same provider		Yes		Yes	Yes	Yes		Yes				
Lack of regular health system communication re LS		Yes			Yes	Yes						
Surveillance Recommendation Adherence												
Colonoscopy (%; intervals adherent of intervals observed)	100% (4 of 4)	100% (1 of 1)	100% (4 of 4)	100% (2 of 2)	100% (1 of 1)	100% (3 of 3)	100% (3 of 3)	0% (0 of 3)	0% (0 of 2)	50% (1 of 2)	100% (6 of 6)	50% (2 of 4)
Endoscopy (%; intervals adherent of intervals observed)	50% (3 of 6)	INC	100% (1 of 1)	100% (1 of 1)	INC	100% (2 of 2)	100% (3 of 3)	0% (0 of 3)	INC	50% (1 of 2)	100% (2 of 2)	INC
Genetics Visit (%; intervals adherent of intervals observed)	100% (4 of 4)	UNS	100% (3 of 3)	100% (1 of 1)	100% (1 of 1)	80% (4 of 5)	60% (3 of 5)	67% (2 of 3)	UNS	100% (2 of 2)	33% (1 of 3)	UNS
Additional details from qualitative interviews												
Remarks on colonoscopy	Intentionally doing it closer to the 2 year part of their 1–2 year interval	Intentionally doing it closer to the 2 year part of their 1–2 year interval				Intentionally doing it closer to the 2 year part of their 1–2 year interval			Difficult to manage a colonoscopy with a colostomy bag, so they are planning to do every 2–3 years because of burden			
Remarks on colonoscopy and endoscopy coordination								Confusion about coordination of endoscopy or if it is needed? Less on the radar than colonoscopy.		Confusion about coordination of endoscopy or if it is needed? Less on the radar than colonoscopy. Patient concerned if they should alternate years	Intentionally timing endoscopy and colonoscopy on different years, but knew to do it	
Remarks on issues with provider	Decided not to do endoscopy again with a discussion with the provider	Trust themselves over doctor/system reminder				Trust themselves over doctor or system reminders				Provider pushed back saying you shouldn’t get a colonoscopy because of age (older patient, greater than 70); trust themselves over doctor/system reminder	Took control of their own care; GI wanted endoscopy every year, but patient pushed back asking to do it every other year, so they could manage their care better; trust themselves over doctor/system reminder	

## Discussion

We hypothesized that, though KPNW had not implemented UTS for CRC cases until January 2016, the EGAPP recommendation would increase awareness of LS and LS-related malignancies and lead to a decrease in time-to-diagnosis from first LS cancer. We found evidence that the rate of LS diagnosis after a cancer diagnosis improved substantially after the EGAPP recommendations for UTS, which may suggest an overall improvement in LS awareness. However, we also identified important care gaps for patients diagnosed with LS within an integrated care system. Since updated UTS recommendations in 2009 [28, 29], a significantly higher percentage of patients were diagnosed with LS within a year of LS-related cancer diagnosis. However, despite these improvements in time-to-diagnosis, we identified only 74 patients in our system with a LS diagnosis, 47 of whom first had a LS-related malignancy. Assuming a population prevalence of 1 in 440 for LS, in the current KPNW adult membership (~ 465,750 people over age 18), we would expect 1059 adults with LS [34]. This drastic under-identification of patients with LS in our study is unlikely to be solely explained by poor documentation of diagnosis status in the EMR, suggesting the biggest care gap for patients with LS in this healthcare system is very likely to be under-diagnosis, especially in the population of individuals who have not yet had a cancer diagnosis.

Because of the variability in recommendations received and different time periods over which patients were diagnosed (where different guidance may have been in place), we analyzed adherence to the actual recommendations provided to the patient and documented in their EMR. We found that most patients with LS received and followed recommendations related to colonoscopy. Most recommended intervals for colonoscopy were at least as often as every 2 years, consistent with current NCCN guideline intervals of 1–2 years [10]. Past studies have reported that a personal history of CRC is a significant predictor of adherence to colonoscopy in LS [20]. In our population, the rate of per-interval adherence to colonoscopy among patients with a prior CRC diagnosis (86%) was higher than those without (73%), though this finding did not reach statistical significance likely due to sample size. Patients with LS and no previous CRC diagnosis may benefit from additional encouragement to follow colonoscopy recommendations. Recommendations related to surveillance for other common LS cancers, such as gastric, endometrial, ovarian, and urinary tract cancer, were less commonly documented in patient charts. However, the penetrance of these cancers varies by genetic variant, and some guidelines have limited which genetic variants warrant these recommendations. Additionally, guideline recommendations have changed over time, some recommendations vary by genetic variant, not all recommendations have equal strength of evidence noted in guidelines, and some guidance uses less stringent language (e.g., “may be considered at the provider’s discretion”) [35]. As such, this may have influenced reduced provision of these recommendations by providers for certain patients. Patients who received extracolonic surveillance recommendations were overall less adherent to these recommendations than they were to colonoscopy. Because we only abstracted provider recommendations from the chart as documented, it is possible that the way the provider framed recommendation statements for extracolonic surveillance to the patient may have impacted patient adherence behaviors (e.g., if the provider told the patient of the lower sensitivity of gynecological surveillance or otherwise de-emphasized its importance). Less than half of eligible women elected to have risk-reducing hysterectomy or BSO after diagnosis of LS. Among those women, 47% were under age 45 at time of chart abstraction, indicating that they could be delaying surgery until childbearing is completed. The remaining patients may have made an individualized decision against or were not advised to have risk-reducing surgery.

Because the variability we observed in recommendations across patients can be explained in part by changes in NCCN and other professional organization guidelines over time, it is important to evaluate if patients receive regular updates to their recommendations. Initial recommendations received by the patient reflect the version in place when the patient received their care recommendations, and few (12%) patients ever received recommendation updates. Despite the fact that guidelines vary and are frequently updated, only 73% of the patient population received recommendations to check back with medical genetics during their ongoing care to receive up-to-date care guidance. Further, though we followed patients for up to 14 years after LS diagnosis, only nine patients had any updates to their surveillance and care recommendations documented in their chart. There is a clear need for routine updates to care recommendations that reflect current guidelines, and a need to standardize LS-related care within health systems.

The care gaps we identified highlight the need for easy-to-implement improvements to care coordination and standardization for this population. In interviews, providers and patients provided concrete suggestions for addressing these gaps, including: 1) clear documentation of the LS diagnosis in the medical record; 2) links to recommended surveillance in an easily accessible section of the EMR; 3) development of automated reminder prompts (email, phone, letter, text) for both patients and providers about upcoming or overdue LS-related surveillance; and 4) establishment of proactive, consistent outreach from medical genetics to review patients’ care and surveillance recommendations against current standards. These suggestions could improve the consistency of recommendations given to patients with LS and improve patient follow up, especially for extracolonic risk-reduction measures.

### Limitations

Our study population was limited to one integrated care system and examined adherence to surveillance care requiring expensive and regular procedures; all patients in this population were members with insurance and access to care. As such, members of the population faced relatively few barriers to adherence. Patterns might differ in non-integrated healthcare systems or in under or uninsured populations, further highlighting the need for evaluation of care coordination in other settings. Due to the lack of a LS-specific diagnostic code, it is possible that this study under-identified patients with a genetic diagnosis of LS in our study population. In particular, it was difficult to identify patients diagnosed with LS prior to enrollment at KPNW. The difficulty identifying LS patients further highlights the need for uniform documentation in the EMR and a specific diagnostic code for this condition. Additionally, our decision to include incomplete intervals in our adherence analysis only if adherence was observed may have slightly inflated our estimates of adherence. Our interview sample size was also relatively small and may not have reflected the full range of patient/provider experiences. Finally, because we relied on recommendations documented in the medical record, it is possible that patients and providers had conversations and made mutual decisions that would affect our measures of adherence that were not recorded.

## Conclusions

Patients with LS were likely to receive and follow recommendations for CRC surveillance. The recommended types and frequency of extracolonic LS-related surveillance varied, and patients were less adherent overall to these recommendations. Patients were unlikely to receive updates to their care recommendations. Both patients and providers felt that care coordination and reminder prompts could be improved. Providers noted the lack of clear documentation of the LS diagnosis and LS recommendations in the medical record as a barrier to care delivery. Improved care coordination and clearer documentation of the LS diagnosis in the EMR is needed.

## Data Availability

The datasets generated and/or analyzed during the current study are not publicly available due to privacy and ethical restrictions but are available from the corresponding author on reasonable request.
